# Characteristics of In-Vitro Starch Digestibility in Wheat Bread with Arabinoxylans, Baked Using Sourdough or Postponed Baking Methods

**DOI:** 10.3390/molecules30081722

**Published:** 2025-04-11

**Authors:** Angelika Bieniek, Krzysztof Buksa

**Affiliations:** Department of Carbohydrate Technology and Cereal Processing, University of Agriculture in Krakow, Balicka 122, 30-149 Krakow, Poland; angelika.bieniek@student.urk.edu.pl

**Keywords:** wheat bread, arabinoxylan, starch digestion, sourdough, postponed baking

## Abstract

The aim of this study was to characterize in vitro digestion of wheat breads baked with sourdough or the postponed baking method without and with arabinoxylan (AX) of different molar mass. The influence of the AX share on the rate of starch digestion, the molar mass of resistant starch (RS) and the pasting characteristics of crumb suspensions of breads baked by the sourdough and postponed baking methods were investigated. Sourdough wheat breads were characterized by contents of very slowly digestible starch (DS) of 1.3% and RS of 1% higher in the crumb, compared to breads baked by the postponed baking method. In the crumb of sourdough breads, after storage for 1 and 3 days, in all variants of the samples (especially with the 2% share of high molar mass AXs), the content of the rapidly digested starch (RDS) fraction decreased, the content of the slowly digestible (SDS) and DS fractions did not change significantly, while the content of the RS fraction increased. In addition, the RS fraction present in the crumb of sourdough breads was generally characterized by a lower molar mass than the RS isolated from the crumb of breads baked by the postponed baking method. The crumb of wheat breads baked using sourdough was characterized by higher viscosity, compared to those baked by the postponed baking method.

## 1. Introduction

Starch is the most abundant component of cereal grains, with a content of 56–90% [1,2,3,4]. The structure and type of starch affect the glycemic index of a food product. After consuming the rapidly digestible starch fraction (RDS), blood glucose levels in the human body increase rapidly. This can be disadvantageous, especially for people struggling with diabetes. The slowly digestible fraction of starch (SDS) is digested slowly but reaches complete degradation in the small intestine. In general, the starch fraction of SDS is considered beneficial for the slow and prolonged release of glucose after ingestion. Resistant starch (RS) is the fraction, which is not digested in the small intestine, but can be fermented in the large intestine [5,6]. The crumb of bread contains RS fraction, which can form ungelatinized starch grains, retrograded starch, as well as starch in the form of amylose–lipid complexes [7,8,9,10]. The previous literature has distinguished a fraction of very slowly digested starch (DS), which was determined by up to 24 h of enzyme digestion [11,12].

Improving the quality of bread by decreasing the content of RDS and increasing the content of RS can have a positive impact on reducing the incidence of metabolic disorders and diseases related to carbohydrate metabolism in humans [13].

Previous studies have noted that the method of baking wheat bread had an ambiguous effect on RDS content [14,15,16,17]. It was shown that sourdough wheat bread [14,16] and wheat rolls baked using the postponed baking method [15,17] had a similar or lower RDS fraction content, compared to wheat bread baked using the single-phase method.

The content of the fraction of slowly digested starch (SDS) was lower in sourdough wheat bread [14,16] and higher [15], or similar [17] in wheat rolls baked by the postponed baking method, compared to wheat bread baked by the single-phase method.

Depending on the study, the crumb of sourdough wheat bread had a higher [14] or lower [16] resistant starch (RS) content, compared to bread baked using the single-phase method. On the other hand, the crumb of wheat bread baked using the postponed baking method showed no difference in RS content, compared to bread baked using the single-phase method [17].

During bread storage, the content of RDS decreased, while the content of SDS and RS increased in sourdough wheat bread, compared to wheat bread baked using the single-phase method [16].

The previous literature has shown that the arabinoxylans (AXs) affected the inhibition of starch digestion in wheat bread baked using the single-phase method. This mechanism was explained by the effect of AX on limiting the access of α-amylase to starch molecules due to increased product viscosity or physical barriers. In addition, ferulic acid, which is covalently attached to the AX molecules, may have reduced the activity of the α-amylase enzyme resulting in reduced starch digestibility [18]. It was shown that the content of RDS and SDS decreased, while the RS content increased with the amount of added AX in wheat bread baked using the single-phase method, compared to bread without added AX [18]. However, there has been no research into the role of AX on starch digestibility in sourdough and postponed bread. Decreasing the content of RDS and increasing the content of SDS and RS have been observed using non-starch polysaccharides other than AXs such as oat β-glucan [19], galactomannans [20], hydroxypropylmethylcellulose, carboxymethylcellulose, xanthan gum and pectin [21].

Moreover, previous publications have not investigated the effect of AXs of different molar mass on the in vitro digestion of starch in the crumb of wheat bread baked with sourdough or by the postponed baking method.

The aim of this study was to characterize the different starch fractions obtained as a result of in vitro digestion of wheat breads baked with sourdough and with the postponed baking method without and with addition of AXs of different molar mass.

## 2. Results and Discussion

One of the primary processes associated with bread aging are changes in starch digestibility and the formation of resistant starch. In this study, we investigated the influence of AX with different molar masses obtained by partial enzymatic hydrolysis and cross-linking of AX on the digestibility and properties of starch in the crumb of wheat breads on the day of baking and during storage. The breads were baked using the sourdough method and the postponed baking method.

The effect of 1% and 2% AX share on the rate of starch digestion, expressed as the content of the fraction of rapidly (RDS), slowly (SDS), very slowly (DS) digestible starch and resistant starch (RS) in the crumb of breads baked using the sourdough and postponed baking methods, was studied.

The available literature data on the content of the various fractions of starch that characterize its digestibility have been inconclusive, which may be due to the complexity of the methods used to determine them. The determination of the content of the above-mentioned starch fractions in the crumb of the breads in this study was performed using the enzymatic method according to Megazyme (based on the methods of Goñi et al. [22] and Englyst et al. [23]). This method has been routinely used to determine the content of RS. In addition, this work uses a modification to determine the content of the product of enzymatic hydrolysis, i.e., glucose, by HPLC/RI, instead of the multi-factor sensitive, enzyme-colorimetric method with glucose oxidase. Such a modification allowed us to increase the specificity and precision of the determination of the content of different fractions of starch. The spectrophotometric method is sensitive to the content of substances that affect the activity of glucose oxidase, the pH of the reaction environment, as well as the content of different compounds that absorb light at a wavelength of 505–510 nm, which can lead to underestimation or overestimation of the results.

Table 1 shows the content of the rapid (within 20 min, RDS), slow (within 120 min, SDS) and very slow (within a further 16 h, DS) amylase digested starch fractions and the content of resistant starch (remaining after 16 h digestion, RS) in the crumb of breads baked using the sourdough and postponed baking methods.

The relative proportions of the determined contents of the starch fractions obtained by amylase digestion, i.e., RDS, SDS contents (Table 1) can be considered typical [24]. However, the content of resistant starch (RS) has been difficult to relate to the literature data, as most authors use starch that has not been digested within 120 min as resistant starch [24,25,26,27], while RS has been defined as the starch remaining after 16 h of digestion [22,23]. It should be noted that the duration of amylase action is closely related to enzyme activity, which has been reported incompletely and ambiguously in many studies.

The previous literature has shown that the contribution of hydrocolloids affects the viscosity of the solution, which consequently also delays the starch hydrolysis process, leading to a lower estimated glycemic response [11]. The hydrocolloids in the bread was the reason why the hydrolysis process was prolonged, and the very slowly digested starch (DS) fraction was separated. Very slowly digested starch (DS) is progressive but nevertheless digested in the small intestine, which means it eventually provides glucose to the organism. Including the DS fraction in the RS fraction would overestimate the result.

When comparing the effect of using various dough handling methods (postponed and sourdough methods) on the content of different starch fractions (Table 1, Section 1), we found that the method used did not significantly affect the content of RDS and SDS fractions (*p* < 0.05). However, an overall higher content of DS and RS fractions was observed in the crumb of breads baked with sourdough. Similar correlations have been reported in the literature, and authors indicate that this is caused by the formation of larger amounts of starch fractions that are difficult to access for digestive enzymes (amylases) in the crumb of sourdough bread [14,24,28]. The increase in RS content in the crumb of sourdough bread has often been explained by the formation of limit dextrins due to the action of bacterial amylases on amylopectin molecules [24]. In addition, the second important factor contributing to RS content is starch retrogradation [24]. In general, a higher total starch content has been observed in sourdough breads compared to those baked by the postponed baking method (Table 1). This has been a consequence of the use of different recipes and technologies, which significantly affected the content of particular components of the product.

Statistical analysis (Table 1, Section 2) showed that the use of all three types of AX generally did not affect (*p* < 0.05) the contents of both rapidly (RDS) and slowly (SDS) digested starch. It should be noted that a trend was observed, according to which the addition of AX resulted in an increase in RDS content, especially in sourdough breads, which could be due to the higher addition of water to doughs with AX (Supplementary Table S2) and the higher degree of starch pasting in the crumb of these breads. On the other hand, the share of AX preparations, especially those of a high molar mass (AX_NM and AX_CR) had a significant effect on reducing the content of very slowly digested starch (DS). In addition, a tendency to increase the content of resistant starch (RS) in the crumb of breads with AX was observed. A significant increase in RS content was observed when AX of the lowest molar mass was added. The observed effect of the AXs has been beneficial, as it may contribute to the reduction in postprandial glycemia. The observed correlations can be explained by the higher degree of starch pasting in crumb of breads with AX (especially AX_CR and AX_NM), in which it was necessary to use a higher addition of water. Indeed, the degree of starch pasting in the bread crumb has been limited by the lack of available water in the crumb [29,30], and non-pasted starch is resistant to enzyme action [31]. It has been shown that AXs can compete with starch for water and inhibit granule swelling and expansion, which can affect the content of the various starch fractions. In addition, AXs can not only adsorb on the surface of starch granules via hydrogen bonds, but can also bind to amylose molecules. Furthermore, ferulic acid, which is part of the arabinoxylan molecules, can bind non-covalently to the starch molecule, which will also contribute to changes in the digestibility of the starch molecules [18,32].

Breads baked with AX addition, especially those of high molar mass (AX_NM and AX CR) were characterized by significantly lower starch content, compared to breads that were control samples. This was due to the higher addition of water to the dough and the higher crumb moisture content of breads with AX (Supplementary Table S2). For breads with AX_HYD, despite the larger addition of water to the dough, there was no significant difference in total starch content compared to breads without AX. This was probably due to the higher volume of breads with AX_HYD (Supplementary Table S2), and consequently, a higher surface area of water evaporation during baking and immediately afterwards during cooling.

Breads obtained with sourdough were tested on the day of baking, but also after 1 and 3 days of storage to assess how the bread aging process affects starch digestibility and resistant starch properties.

Table 2 (Section 3) shows the statistical analysis of the effect of storage time on the content of different starch fractions after the digestion of the crumb of breads baked with sourdough.

After bread storage for 1 and 3 days, in all variants of samples (without and with added AX) obtained by the sourdough method, the RDS content in the bread crumb decreased, the SDS content did not change significantly (*p* < 0.05), while the DS and RS content generally increased. The highest RS contents were observed after 3 days of storage. The observed correlations confirm the literature data, which identifies retrogradation of the starch fraction as one of the main causes of bread aging, and this process significantly reduces its digestibility [24]. It should be noted that retrogradation was associated with the loss of water from the crumb of the breads, as evidenced by the observed increasing starch content on subsequent days of storage.

**Figure 1:**
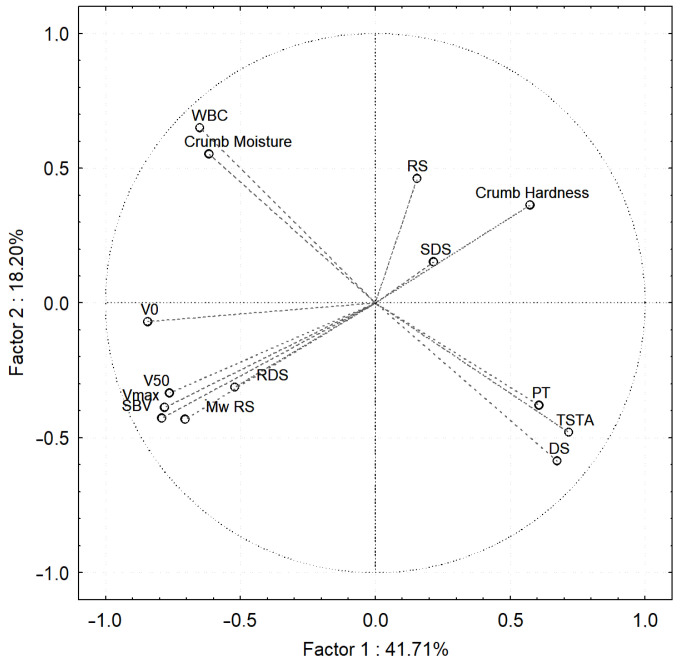
Principal component analysis: the influence of the addition of arabinoxylans with different molar mass on the crumb properties of bread baked by the postponed and sourdough methods, obtained on the day of baking and during bread storage. WBC—water binding capacity; SBV—specific bread volume; M_w_—molar mass of resistant starch; V_0_—initial viscosity; V_max_—maximum viscosity, V_50_—viscosity at 50 °C; PT—pasting temperature; RDS—rapidly digestible starch; SDS—slowly digestible starch; DS—digestible starch; RS—resistant starch; TSTA—total starch.

As a result of Principal Component Analysis (PCA, Figure 1), it was found that as crumb hardness increased, RDS contents decreased, while RS contents increased, which can be explained by the more difficult and slower enzymatic hydrolysis of the crumb of breads with high bread crumb hardness. In addition, the crumb with high moisture content had a small amount of very slowly digested starch (DS). The observed correlations were probably related to the different degrees of starch pasting in the crumb of the breads, depending on the water content.

Including all the variants studied, no significant correlations were found between the content of RDS and SDS and the moisture content of the crumb of the breads. It should be noted that such correlations occurred within particular dough handling technologies, but additional research is needed to clarify their mechanisms, which is beyond the scope of this publication.

To clarify the role of retrogradation in bread aging and to test the effect of the additives and baking technology used on RS molecules from the crumb of breads obtained on the day of baking, as well as during storage, resistant starch was extracted and its molar mass determined by SEC chromatography. The distributions of molar masses of resistant starch extracted from the crumb of breads obtained by sourdough and postponed baking methods are shown in Supplementary Figures S1, S2 and S3, respectively.

The fraction in the range of 40,000 g/mol was identified as resistant starch (RS). The area under the peaks expressed the approximate polysaccharide content, which was dependent on the type of sample tested.

Based on the elution profiles obtained, the molar mass distributions were calculated, and the molecular parameters of RS isolated from the crumb of the tested breads were determined, i.e., weight average molar mass (M_w_), number average molar mass (M_n_) and dispersity (Đ). The molecular parameters of RS are shown in Table 3 and Table 4.

In the available literature, information about the molar mass of RS in bread has been fragmentary, which makes it impossible to compare the results obtained. In general, the molar mass values presented in Table 3 were of the same order as those presented in the literature on rye and wheat starch [33,34].

Analyzing the effect of the applied dough handling technology on the molecular properties of RS (Table 3), it was found that RS present in the crumb of breads prepared with sourdough had a lower (*p* < 0.05) molar mass (with the exception of bread with 2% AX_NM) than RS isolated from the crumb of breads baked by the postponed baking method.

Statistical analysis of the molecular parameters of RS isolated from the crumb of breads baked by both the sourdough and postponed baking methods (Table 3) showed that the addition of AX_HYD and AX_CR did not significantly affect the molar mass of isolated RS. However, the addition of the AX_NM preparation, especially at the 2% level, caused the isolated RS to have a higher molar mass (*p* < 0.05), compared to the resistant starch isolated from the crumb of control breads (without AX addition). AX_NM significantly affected the addition of water to the dough by which AX_NM could influence the increase the degree of starch pasting. Moreover, the AX_HYD formulation could effectively interact with starch molecules and promote the formation of RS (especially by large starch molecules), in contrast to the very large arabinoxylan molecules from the AX_CR preparation.

In addition, the RS content of all bread variants increased during storage (Table 2), while the molar mass of resistant starch increased after 1 day of storage (*p* < 0.05), except for bread with a 2% share of AX_NM (Table 4). As a result of Principal Component Analysis (PCA, Figure 1), RS of relatively low molar mass was observed in the crumb with high hardness.

Higher RS dispersity values were observed as a result of postponed baking technology compared to sourdough bread. The influence of the application and type of AX on RS dispersity values was inconclusive. During bread storage, an increase in RS dispersity values was noted, indicating that starch molecules of different sizes were involved in the formation of resistant starch.

Amylographic analysis of the bread crumb has been used by some researchers to assess the degree of starch pasting, the interaction between starch molecules, as well as the interaction of starch molecules with other dough components during bread baking and the retrogradation that occurs after baking [35,36,37,38,39]. Most of the research has been about typical wheat bread. To date, amylographic analysis has not been used to evaluate the crumb of sourdough breads and breads baked by the postponed baking method with AXs of different molar masses. To more precisely investigate the effect of added AX on starch properties and the transformations and interactions of wheat dough components, the bread crumbs were frozen and lyophilized, and then the suspensions of lyophilized crumbs of each bread were examined in an amylograph. The amylographic characteristics of the suspensions of lyophilized bread crumbs obtained from breads baked by the sourdough method on the day of baking and after 1 and 3 days of storage and the postponed baking method are shown in Supplementary Figures S4, S5 and S6, respectively.

Despite the use of a different flour, differences in dough handling and baking technology, a different methodology for preparing crumb samples for analysis (including the use of lyophilization) and a different concentration of suspensions, the amylographic characteristics obtained (Figures S4–S6) showed some similarity to the amylographic curves obtained in wheat bread crumb studies presented in the literature [35,36,37,38,39]. The amylographic curves of the bread crumb differed significantly from typical amylographic characteristics obtained when testing starches or flours. Similarly to the case of bread crumbs baked using the traditional method [35,36,37,38], during the amylographic analysis of crumb samples of sourdough and postponed baked wheat breads (which are control samples), the typical increase in viscosity during heating was not observed, and the viscosity of the suspensions increased only when the temperature reached 90 °C. However, an increase in viscosity was observed in samples with AX during heating, and its intensity depended on the variant tested. During and after the cooling of the suspensions, the viscosity of all samples increased rapidly, with the intensity depending on the baking technology and the type of AX used.

On the basis of the amylograms obtained, parameters such as: initial viscosity, which was recorded when the suspensions were stirred at 30 °C; initial temperature of pasting (PT); maximum viscosity, which was determined when the bread crumb suspensions were heated in the amylograph; final viscosity, which was recorded after the bread crumb suspensions were cooled to 50 °C, was determined. The values of the amylographic parameters were subjected to statistical analysis and the results are presented in Table 5 and Table 6.

The amylograph-determined initial viscosity values of the crumb suspensions of the breads (Table 5) were not large but showed significant variation. A statistical analysis showed that the addition of all types of AX resulted in an increase (*p* < 0.05) in the initial viscosity (Table 5). The effect of increasing initial viscosity values was similar for all types of preparations. The observed correlation can be explained by the influence of AX on viscosity. It was observed that the type of dough handling technology used had a significant effect on the values of initial viscosity, as the crumb of breads baked by the postponed method was characterized by higher values of this parameter, compared to the crumb of breads baked on sourdough. This was probably a consequence of the relatively low pH of the dough and the acid hydrolysis that occurs under such conditions, both of AX and starch, during the acidification of the flour and subsequent baking phases. The lower viscosity of crumb suspensions of bread baked with sourdough may also have been influenced by changes in protein structure in the acidic environment. Indeed, partial hydrolysis of polysaccharides present in flour, as well as structural changes in proteins, occurring in an acidic environment during baking, are widely reported in the literature [40].

Analyzing the initial viscosity values determined for the crumb obtained from the breads on the day of baking and during subsequent days of storage (Table 6), it was found that the initial viscosity values generally decreased during storage, which was also observed in similar studies of the crumb of traditional breads [40]. The mentioned correlation can be related to the formation of strong, hardly water-soluble starch aggregates in the crumb of the breads, which did not contribute significantly to the increase in viscosity of the suspensions when measured at 30 °C.

During the heating of the crumb suspensions of the breads in the amylograph, the viscosity increased (Figures S4–S6), and this result was interpreted as a re-pasting of that part of the starch which, due to the limited availability of water in the dough, did not gelatinize during the baking of the bread in the oven [30,35,36,37,38]. As shown in Table 5 (Section 1), a significantly higher starch pasting temperature was observed in the crumb of sourdough bread compared to bread baked by the postponed baking method. Based on the statistical analysis, it was concluded (Table 5) that, generally, the addition of all types of AXs, both to bread with sourdough and baked by the postponed baking method, resulted in a decrease in the pasting temperature (PT) of ungelatinized and partially gelatinized starch in the crumb of the breads. This effect was particularly evident when AX_HYD and AX_CR were present in the samples. In addition, a decrease (*p* < 0.05) in the pasting temperature of starch present in the crumb of breads obtained on subsequent days of storage (Table 6, Section 3) was observed. The observed effect may support the hypothesis that the recrystallization of starch (mainly amylose) occurred during storage of the breads, as also indicated by other authors [35,36,37,38,41,42,43].

Based on the results of the analyses presented in Table 5, the effect of using AX for baking on the maximum viscosity of the crumb suspension was not conclusive and depended on the dough handling technology. The use of AX_CR resulted in a significant increase (*p* < 0.05), and the use of AX_HYD resulted in a significant decrease in the maximum viscosity value, while the use of AX_NM did not significantly affect this parameter. The observed influence can be explained by the fact that the viscosity of the bread crumb was proportionally affected by the molar mass of AXs. The observed increase in viscosity during the heating of the suspension from 30 °C to 92 °C, can be interpreted as the starch pasting of the part of the starch that did not paste during baking, due to the limited access of water in the dough (necessary for the complete process). AXs, depending on their molar mass, inhibited starch pasting to different degrees during baking, resulting in more intense pasting and increased viscosity during analyses of bread crumb suspensions in the amylograph. AX of a high molar mass, especially AX_CR, effectively hindered the access of water to the starch granules.

As noted by Yasunaga et al. [35], the amylographic evaluation of changes in the viscosity of crumb obtained from bread during storage allows assessment of the degree of aging of bread. Based on the conducted amylographic measurements, a general trend was observed, after heating the crumb suspensions of all breads, the maximum viscosity (Table 6) was the highest on the day of baking and decreased after 1 and 3 days of storage. This may be another indication of the formation of starch aggregates during bread storage, characterized by strong bonds between its linear molecules (especially amylose chains), which did not dissolve in hot water and did not significantly contribute to the increase in the viscosity of the suspensions [34,35,36,37,38,44,45,46]. A principal component analysis (PCA, Figure 1) including all results obtained from bread crumb analyses showed that the bread crumb with high hardness was characterized by low values of maximum viscosity, viscosity after cooling, and low initial viscosity of suspensions.

The registered viscosity values after cooling the suspensions to 50 °C correlated (Figure 1) with the maximum viscosity values for the corresponding samples, but were significantly higher (Table 6). In addition, on all amylograms, the occurrence of characteristic “bumps” was observed during the cooling of crumb suspensions, which have also been reported in previous studies [36,37,38]. The observed increase in viscosity after cooling and the presence of “bumps” on the amylograms may indicate strong interactions occurring between crumb components. Previous studies have indicated the influence of lipid substances on the intensity of interactions between starch molecules [35,36,37,38]. Based on the results from the present study, it was concluded that AXs can also have a significant effect on starch interactions, which expands the knowledge of factors affecting bread aging processes.

The results obtained from amylographic determinations of the crumb of the breads correlated with the results of starch digestibility. A principal component analysis (PCA, Figure 1) showed that the crumb of breads with high initial viscosity, maximum viscosity and viscosity after cooling suspensions were characterized by high RDS content and low SDS and RS content. In addition, a positive correlation was found between the temperature of pasting and DS content. The very slowly digested starch fraction (DS) probably consists of starch from small granules that did not fully paste during bread baking and such granules pasted at a higher temperature when crumb suspensions were measured in the amylograph.

The observed effects are a consequence of the different degree of aggregation of starch molecules described in this paper, due to the absence or presence of non-starch polysaccharides, which affected the viscosity and susceptibility of starch to amylase digestion. The presence of similar relations was suggested in their study by Yasunaga et al. [35]. It should be noted that the presented correlations can be used to predict the digestibility of starch in the crumb of bread based on the amylographic analysis of its suspensions.

## 3. Materials and Methods

Wheat breads were baked using wheat flour type 750 (PZZ Krakow, Poland). Starter cultures LV2 (SAF, LEVAIN Lesaffre, Marcq-en-Barœul (Nord), France) were used for sourdough bread. Other baking additives included freeze-dried yeast *Saccharomyces cerevisiae* (Lesaffre, France) and non-iodized salt (NaCl, Avantor Performance Materials Poland S.A. from Gliwice, Poland).

Arabinoxylans were isolated from rye flour of the Amilo variety (Danko, Choryń, Poland) produced by laboratory method.

### 3.1. Isolation and Modification of Preparations

The water-soluble AXs were isolated from rye flour and modified according to the methods of Buksa et al. [47].

#### Isolation and Modifications of Arabinoxylans

The procedures of isolation and the modification of arabinoxylans were presented in previous papers [48,49]. The modification of arabinoxylans was performed using cross-linking and partial enzymatic hydrolysis. A detailed description of the modification method can be found in the Supplementary file: “Supplementary Information—AX Modification”. In short, to obtain a preparation of cross-linked arabinoxylans, the obtained lyophilizate was treated with hydrogen peroxide (1 μg/g AXs) and peroxidase (5 U/g AXs). The AX precipitate was centrifuged and washed to remove all water from the sample, and then dried at 50 °C for 2 h until the cross-linked AX was prepared. To obtain a preparation of partially hydrolyzed arabinoxylans, the lyophilized product was treated with the enzyme xylanase (endo-β-1,4-xylanase) of *Thermomyces lanuginosus* (Merck Life Science Sp.z.o.o., an affiliate of Merck KGaA, Darmstadt, Germany). The AX precipitate was centrifuged, washed, and then dried at 50 °C for 2 h until it became a partly hydrolyzed AX preparation. Detailed characteristics of the obtained preparations were discussed in [48,49] and basic characteristics of arabinoxylan preparations are summarized in Supplementary Table S1. The non-modified AX preparation was denoted as AX_NM, the preparation of cross-linked AX was denoted as AX_CR and the preparation of partly hydrolyzed AX was denoted as AX_HYD.

### 3.2. Baking Breads

#### 3.2.1. Baking Sourdough Wheat Bread with Arabinoxylans

Sourdough wheat bread was baked according to the method of Bieniek and Buksa [48]. In short, we added and water to wheat flour LV2 starter culture (obtaining a dough yield of 220%). After mixing, the sourdough samples were incubated in a fermentation chamber at 30 °C for 24 h. In the case of the control dough, after sourdough preparation, a new portion of wheat flour was added to the sourdough to obtain a 25% proportion of flour from the sourdough in the whole dough, we added yeast 2.5%, salt 1.8% and water in the amount determined using the farinograph (dough with a consistency of 500 BU, determined according to ICC-Standard No. 115/1 [50]). In order to test the influence of AX on sourdough, wheat AX preparations of 1% and 2% by weight of flour were added in place of flour. All dough samples were mixed, placed in a baking pan and placed in the fermentation chamber for 40 min and finally baked.

#### 3.2.2. Baking Wheat Bread Using the Postponed Baking Method with a Share of Arabinoxylans

The breads were baked according to Buksa et al. [51]. According to the base recipe, the dough was made from wheat flour, water, 1.8% salt and 3.5% yeast. In order to test the influence of AX, AX preparations of 1% and 2% by weight of flour were added in place of flour. In the farinograph mixer, doughs of equal consistency of 500 BU (determined according to ICC-Standard No. 115/1 [50]) were made from the tested flours, from which, after shaping the pieces and fermentation (60 min), the breads were baked. The dough pieces were placed in an oven preheated to 160 °C; the temperature gradually increased to reach 190 °C and was kept at 190 °C for 3 min, without allowing the crust to color. The partly baked breads were then cooled and frozen until a temperature of −23 °C was reached inside the loaves. The frozen breads were stored in the freezer for 2 weeks. After defrosting, the breads were re-baked at 230 °C.

### 3.3. Bread Characteristics

Detailed characteristics of sourdough wheat breads and wheat breads baked using the postponed baking method was presented and discussed in detail in previous works. Supplementary Table S2 contains the most important parameters of baked breads [48,49].

#### 3.3.1. Determination of Crumb Digestion Dynamics and Resistant Starch Content in the Crumb of Breads

The determination was performed on the crumb of breads baked using the sourdough method (obtained on the day of baking and after 1 and 3 days of storage) and breads baked using the postponed baking method (obtained on the day of baking). The crumb digestion dynamics of the breads were investigated by determining the content of rapidly (RDS), slowly (SDS) and very slowly (DS) digested starch, using a modified method according to the Megazyme resistant starch test (AACC method 32–40), based on the methods of Goñi et al. [22] and Englyst et al. [23]. For the determination, 4 mL of 100 mM sodium maleate buffer (pH 6.0) containing the enzymes α-amylase (porcine pancreatic α-amylase, Sigma-Aldrich, Saint Louis, MO, USA) and amyloglucosidase (Megazyme, Bray, Ireland) were added to test tubes containing 250 mg dry weight of homogenized crumb. The closed tubes were shaken horizontally in a water bath at 35 °C (200 beats per minute). At time intervals of 0, 20, 60, 120 and 960 min, 100 uL of hydrolysate was collected, to which 500 μL of 99% ethanol was immediately added to inactivate amylolytic enzymes. Inactivated samples were diluted 1:1 with deionized water, centrifuged (21,000× *g*; 10 min) and 20 μL injected into an HPLC/RI (Knauer Wissenschaftliche Geräte GmbH, Berlin, Germany), according to Buksa et al. [47], to determine the glucose content and calculate the content of rapidly (RDS), slowly (SDS) and very slowly (DS) digested starch. The fraction of very slowly digestible starch (DS) was determined based on the studies Santamaria et al. [11]. RDS, SDS and DS content were calculated using the following equations:

RDS = (Glc20 − FGlc) × 0.9,

SDS = (Glc120 − Glc20) × 0.9,

DS = (Glc16h − RDS − SDS) × 0.9,

where

-Glc20, Glc120 and Glc16h are the glucose contents determined after 20, 120 min and 16 h digestion, respectively.

-FGlc is the glucose content determined after 0 min digestion.

The glucose (Glc) content was multiplied by a factor of 0.9, which is calculated from the molar mass of starch monomer/molar mass of glucose (162/180 = 0.9).

#### 3.3.2. Determination of Resistant Starch in the Crumb of Breads

The determination of resistant starch (RS) content was conducted using the resistant starch assay according to Megazyme (method 32–40, AACC), with an author’s modification. Analogous to the determination of digestion dynamics (Section 3.3.1), 4 mL of 100 mM sodium maleate buffer (pH 6.0) containing the enzymes α-amylase and amyloglucosidase was added to test tubes containing 100 mg dry weight of homogenized crumb. The sealed tubes were shaken horizontally in a water bath at 35 °C (200 beats/min) for 16 h. The reaction was stopped by adding ethanol (99.8%), and the RS was extracted as a precipitate after centrifugation (2000× *g*; 10 min). The precipitate containing RS was then dissolved in 50% ethanol, centrifuged (2000× *g*; 10 min) and the supernatant discarded by decantation. Washing the precipitate with 50% ethanol was performed twice. In the next step, the precipitate containing RS was dissolved in 2 mL 2 M KOH by energetic stirring in an ice-water bath on a magnetic stirrer. The solution was neutralized by adding 8 mL of 1.2 M acetate buffer. RS was then hydrolyzed to glucose by adding 100 μL of amyloglucosidase and incubating at 50 °C for 20 min. After cooling, the hydrolysate was centrifuged (2000× *g*; 10 min) and 20 μL of the supernatant was injected into an HPLC/RI kit (Knauer Wissenschaftliche Geräte GmbH, Berlin, Germany) for the determination of glucose and calculation of resistant starch (RS). The chromatographic setup was equipped with a Sugar SP-0810 column (Shodex, Japan). The column temperature during the analyses was 70 °C. Deionized water was used as an eluent, with a flow rate of 1 mL/min. The glucose content was calculated from a calibration curve obtained from analyses of glucose standard solutions at concentrations of 0–2 mg/mL. The RS content of the samples was calculated using Clarity software (ver. 4.0.1.700, DataApex, Prague, Czech Republic) as the determined glucose content multiplied by the polymerization factor (Glc × 0.9).

#### 3.3.3. Determination of the Molar Mass of Resistant Starch in the Crumb of Breads

The determination of the molar mass distributions of resistant starch (RS) was performed using the HPSEC/RI method (Knauer Wissenschaftliche Geräte GmbH, Berlin, Germany) according to Buksa [33]. For the determination, resistant starch was isolated from the crumb of the breads baked using the sourdough method (obtained on the day of baking and after 1 and 3 days of storage) and breads baked using the postponed baking method (obtained on the day of baking). The first step of the determination was carried out exactly as for the determination of RS content (Section 3.3.2). After washing the precipitate twice with 50% ethanol, the precipitate (resistant starch) was dried at 50 °C for 60 min. Then, 1 mL of DMSO was added to the dried precipitate, the precipitate was dissolved at 70 °C for 24 h using a magnetic stirrer, and the samples were centrifuged (21,000× *g*; 10 min) and 100 µL of the supernatant was injected into the HPSEC/RI. RS molar mass analysis was performed on an HPSEC/RI consisting of OHpak columns SBG, SB804 and SB802 (Shodex, Tokyo, Japan) connected in series and an RI detector. The separation was conducted at 60 °C. An aqueous solution of 100 mM NaNO_3_ at a flow rate of 0.6 mL/min was used as the eluent. The calibration of the HPSEC system was performed using solutions of glucose, maltose and pullulan standards of known molar mass (P-5, 10, 100, 400 and 800; Shodex Standard, Macherey-Nagel). The molar mass distributions were used to calculate the apparent molar mass (M_w_, M_n_) and dispersity (Ð = M_w_/M_n_) of RS using Eurochrom (Knauer, Berlin, Germany) and Clarity (DataApex) software ver. 4.0.1.700.

#### 3.3.4. Measurements of Bread Crumb Viscosity During Heating in an Amylograph

The determination was performed on the crumb of breads baked on sourdough and with the postponed baking method collected 2 h after baking and after 1 and 3 days of storage. The bread crumb was frozen immediately after sampling and then lyophilized. The resulting lyophilized samples were crushed in a mortar and sieved through a 0.5 mm mesh sieve before weighing. Viscosity measurements of lyophilized bread crumb samples obtained on the day of baking and after 1 and 3 days of storage were conducted in a Micro Visco Amylograph (Brabender, Anton Paar GmbH, Graz, Austria) using the method according to Buksa et al. [34] with modifications. The viscosity of 11% (*w*/*w*, d.m.) lyophilized bread crumb suspensions was tested during the following temperature profile: an initial temperature of 30 °C was observed for 2 min, then the samples were heated to 92 °C and held at this temperature for 5 min, followed by cooling to 50 °C and holding at this temperature for 5 min. The heating and cooling rate was 7.5 °C/min and the measuring cylinder rotated at 300 rpm.

### 3.4. Statistical Analysis

All analyses, unless otherwise stated, were performed in at least three replicates. MS Excel 2007 (Microsoft, Redmond, WA, USA) and StatSoft Statistica v. 9.0 (StatSoft, Inc., Tulsa, OK, USA) software were used for the statistical analysis of the results. Statistical analysis included the calculation of mean values and standard deviation, analysis of variance, NIR test with a significance level of *p* < 0.05. Additionally, to evaluate the selected data, Principal Component Analysis (PCA) was applied.

## 4. Conclusions

Sourdough wheat breads were characterized by a higher content of the very slowly digested starch DS fraction and RS fraction in the crumb, compared to breads baked by postponed baking method. The use of all types of AXs in wheat breads did not influence the contents of both rapidly (RDS) and slowly (SDS) digested starch. In the crumb of sourdough bread, after storage for 1 and 3 days, in all variants of the samples (especially with the 2% share of AX_NM and AX_CR), the content of the RDS fraction decreased, the content of the SDS and DS fractions did not change significantly, while the content of RS increased. The RS present in the crumb of sourdough breads was characterized by a lower molar mass (with the exception of bread with 2% AX_NM) than the RS isolated from the crumb of breads baked by postponed baking method. The 2% share of AX_NM had the highest effect on increasing the molar mass of RS, compared to bread without AX. The crumb of sourdough baked wheat breads treated with re-pasting in the amylograph had a higher maximum viscosity and the viscosity after cooling to 50 °C, compared to the crumb of breads baked by the postponed baking method. The differences in the viscosity of the crumb of breads baked by the sourdough and postponed baking methods were due to the different content of ungelatinized and partially gelatinized starch.

## Figures and Tables

**Table 1:** Total starch content and its different fractions in the crumb of wheat bread with arabinoxylan (AX) preparation baked with sourdough and the postponed baking method on the day of baking.

	Level of	Technology **	Share [%]	RDS *** [%]	SDS *** [%]	DS *** [%]	RS *** [%]	TS *** [%]
Technology	SD			4.1 ± 0.5 ^a^	9.0 ± 0.9 ^a^	32.4 ± 3.1 ^b^	7.6 ± 0.6 ^b^	39.9 ± 3.0 ^b^
Technology	PB			4.4 ± 0.4 ^a^	8.9 ± 1.7 ^a^	31.1 ± 1.3 ^a^	6.6 ± 0.7 ^a^	37.7 ± 1.5 ^a^
Preparation	Control			4.1 ± 0.6 ^a^	9.8 ± 0.4 ^a^	35.1 ± 2.7 ^c^	6.3 ± 0.5 ^a^	41.4 ± 3.2 ^b^
Preparation	AX_NM *			4.2 ± 0.5 ^a^	8.8 ± 1.0 ^a^	30.6 ± 1.2 ^a^	6.8 ± 0.6 ^ab^	37.4 ± 1.5 ^a^
Preparation	AX_HYD *			4.4 ± 0.4 ^a^	8.9 ± 1.2 ^a^	32.9 ± 1.7 ^b^	7.6 ± 0.6 ^c^	40.5 ± 2.3 ^b^
Preparation	AX_CR *			4.2 ± 0.5 ^a^	8.7 ± 1.9 ^a^	30.0 ± 1.3 ^a^	7.2 ± 1.0 ^ab^	37.2 ± 1.1 ^a^
Share [%]	0			4.1 ± 0.6 ^a^	9.8 ± 0.4 ^b^	35.1 ± 2.7 ^b^	6.3 ± 0.5 ^a^	41.4 ± 3.2 ^b^
Share [%]	1			4.3 ± 0.6 ^a^	8.1 ± 1.4 ^a^	31.4 ± 2.0 ^a^	7.3 ± 0.9 ^b^	38.7 ± 2.3 ^a^
Share [%]	2			4.3 ± 0.4 ^a^	9.5 ± 1.0 ^b^	30.9 ± 1.8 ^a^	7.0 ± 0.7 ^ab^	38.0 ± 2.2 ^a^
Sample×Technology	Control	SD	0	3.7 ± 0.5 ^a^	10.0 ± 0.6 ^b^	37.2 ± 0.8 ^d^	6.7 ± 0.1 ^ab^	43.9 ± 0.9 ^d^
Sample×Technology	AX_NM *	SD	1	4.0 ± 0.9 ^a^	8.2 ± 0.0 ^ab^	31.4 ± 0.1 ^abc^	7.3 ± 0.1 ^ab^	38.7 ± 0.3 ^abc^
Sample×Technology	AX_NM *	SD	2	4.6 ± 0.3 ^a^	8.6 ± 0.3 ^ab^	31.0 ± 1.8 ^abc^	7.0 ± 0.2 ^ab^	38.0 ± 2.0 ^ab^
Sample×Technology	AX_HYD *	SD	1	4.3 ± 0.5 ^a^	8.9 ± 01.5 ^ab^	34.8 ± 0.4 ^cd^	8.2 ± 0.3 ^ab^	43.0 ± 0.6 ^cd^
Sample×Technology	AX_HYD *	SD	2	4.5 ± 0.4 ^a^	8.4 ± 1.5 ^ab^	34.0 ± 0.9 ^bcd^	8.0 ± 0.1 ^ab^	42.0 ± 0.7 ^bcd^
Sample×Technology	AX_CR *	SD	1	3.7 ± 0.6 ^a^	9.3 ± 0.2 ^b^	29.1 ± 1.3 ^a^	8.3 ± 0.3 ^b^	37.4 ± 1.0 ^ab^
Sample×Technology	AX_CR *	SD	2	4.1 ± 0.0 ^a^	9.5 ± 0.1 ^b^	28.9 ± 0.7 ^a^	7.4 ± 0.1 ^ab^	36.3 ± 0.6 ^a^
Sample×Technology	Control	PB	0	4.5 ± 0.0 ^a^	9.7 ± 0.4 ^b^	33.0 ± 1.7 ^abc^	6.0 ± 0.6 ^a^	38.9 ± 2.2 ^abc^
Sample×Technology	AX_NM *	PB	1	4.5 ± 0.5 ^a^	8.7 ± 1.1 ^ab^	30.1 ± 1.3 ^ab^	6.7 ± 0.7 ^ab^	36.8 ± 0.6 ^a^
Sample×Technology	AX_NM *	PB	2	3.9 ± 0.3 ^a^	9.9 ± 1.4 ^b^	30.0 ± 1.5 ^ab^	6.1 ± 0.4 ^ab^	36.1 ± 1.9 ^a^
Sample×Technology	AX_HYD *	PB	1	4.7 ± 0.8 ^a^	7.9 ± 0.7 ^ab^	32.0 ± 0.3 ^abc^	7.1 ± 0.6 ^ab^	39.1 ± 0.3 ^abcd^
Sample×Technology	AX_HYD *	PB	2	4.3 ± 0.3 ^a^	10.1 ± 0.1 ^b^	31.0 ± 0.4 ^abc^	6.9 ± 0.1 ^ab^	37.9 ± 0.3 ^ab^
Sample×Technology	AX_CR *	PB	1	4.4 ± 0.6 ^a^	5.8 ± 0.5 ^a^	31.2 ± 0.9 ^abc^	6.3 ± 1.2 ^ab^	37.5 ± 2.1 ^ab^
Sample×Technology	AX_CR *	PB	2	4.5 ± 0.5 ^a^	10.1 ± 1.3 ^b^	30.8 ± 0.8 ^abc^	6.8 ± 1.2 ^ab^	37.6 ± 0.4 ^ab^

* AX_NM—non-modified AX; AX_HYD—hydrolyzed AX; AX_CR—cross-linked AX. ** SD—sourdough baking method; PB—postponed baking method. *** RDS—rapidly digestible starch; SDS—slowly digestible starch; DS—digestible starch; RS—resistant starch; TS—total starch. Mean values marked with the same letters in particular columns are not statistically significantly different at *p* < 0.05.

**Table 2:** Total starch content and its different fractions in the crumb of wheat bread with arabinoxylan (AX) preparations baked with sourdough on the day of baking and after 1 and 3 days of storage.

	Level of	Day of Storage	Share [%]	RDS *** [%]	SDS *** [%]	DS *** [%]	RS *** [%]	TS *** [%]
Preparation	Control			3.4 ± 0.4 ^a^	10.8 ± 0.8 ^b^	36.4 ± 1.0 ^c^	8.3 ± 1.5 ^ab^	44.7 ± 1.7 ^b^
Preparation	AX_NM *			3.9 ± 0.7 ^b^	9.6 ± 1.2 ^a^	32.4 ± 1.5 ^a^	7.9 ± 1.0 ^a^	40.3 ± 2.1 ^a^
Preparation	AX_HYD *			3.9 ± 0.7 ^b^	8.9 ± 1.0 ^a^	34.6 ± 1.0 ^b^	9.3 ± 1.0 ^b^	44.0 ± 1.3 ^b^
Preparation	AX_CR *			3.4 ± 0.6 ^ab^	9.3 ± 0.7 ^a^	31.2 ± 2.4 ^a^	8.8 ± 1.3 ^ab^	40.0 ± 2.5 ^a^
Share [%]	0			3.4 ± 0.4 ^a^	10.8 ± 0.8 ^b^	36.4 ± 1.0 ^b^	8.3 ± 1.5 ^a^	44.7 ± 1.7 ^b^
Share [%]	1			3.7 ± 0.6 ^a^	9.3 ± 1.2 ^a^	33.2 ± 2.1 ^a^	8.4 ± 1.0 ^a^	41.7 ± 2.7 ^a^
Share [%]	2			3.9 ± 0.8 ^a^	9.2 ± 0.9 ^a^	32.3 ± 2.2 ^a^	8.9 ± 1.4 ^a^	41.2 ± 2.7 ^a^
Day	0			4.1 ± 0.5 ^b^	9.0 ± 0.9 ^a^	32.4 ± 3.1 ^a^	7.6 ± 0.6 ^a^	39.9 ± 3.0 ^a^
Day	1			4.0 ± 0.5 ^b^	9.8 ± 1.0 ^a^	34.0 ± 1.7 ^b^	8.8 ± 1.0 ^b^	42.7 ± 2.4 ^b^
Day	3			3.0 ± 0.4 ^a^	9.7 ± 1.3 ^a^	33.5 ± 2.3 ^ab^	9.5 ± 1.2 ^b^	43.0 ± 2.0 ^c^
Sample×Day	Control	0	0	3.7 ± 0.5 ^abcd^	10.0 ± 0.6 ^ab^	37.2 ± 0.8 ^d^	6.7 ± 0.1 ^a^	43.9 ± 0.9 ^de^
Sample×Day	Control	1	0	3.5 ± 0.3 ^abcd^	10.9 ± 0.3 ^ab^	35.5 ± 1.1 ^cd^	9.1 ± 1.4 ^abcd^	44.6 ± 2.5 ^e^
Sample×Day	Control	3	0	3.2 ± 0.3 ^abcd^	11.7 ± 0.5 ^b^	36.6 ± 0.7 ^d^	9.1 ± 1.4 ^abcd^	45.6 ± 2.2 ^e^
Sample×Day	AX_NM *	0	1	4.0 ± 0.9 ^abcd^	8.2 ± 0.0 ^a^	31.4 ± 0.1 ^abc^	7.3 ± 0.1 ^ab^	38.7 ± 0.3 ^abcd^
Sample×Day	AX_NM *	1	1	4.0 ± 0.2 ^abcd^	9.9 ± 0.6 ^ab^	32.6 ± 2.1 ^abcd^	7.5 ± 1.4 ^abc^	40.1 ± 3.6 ^abcde^
Sample×Day	AX_NM *	3	1	3.5 ± 0.1 ^abcd^	11.2 ± 1.2 ^ab^	33.5 ± 1.2 ^abcd^	8.3 ± 1.4 ^abcd^	41.8 ± 0.2 ^abcde^
Sample×Day	AX_NM *	0	2	4.6 ± 0.3 ^d^	8.6 ± 0.3 ^ab^	31.0 ± 1.8 ^abc^	7.0 ± 0.2 ^ab^	38.0 ± 2.0 ^abc^
Sample×Day	AX_NM *	1	2	4.5 ± 0.4 ^cd^	9.8 ± 1.3 ^ab^	33.3 ± 1.6 ^abcd^	8.4 ± 0.6 ^abcd^	41.7 ± 1.1 ^abcde^
Sample×Day	AX_NM *	3	2	2.8 ± 0.4 ^ab^	9.7 ± 1.2 ^ab^	32.6 ± 1.9 ^abcd^	8.9 ± 0.5 ^abcd^	41.5 ± 1.4 ^abcde^
Sample×Day	AX_HYD *	0	1	4.3 ± 0.5 ^abcd^	8.9 ± 1.5 ^ab^	34.8 ± 0.4 ^cd^	8.2 ± 0.3 ^abcd^	43.0 ± 0.6 ^cde^
Sample×Day	AX_HYD *	1	1	4.4 ± 0.1 ^abcd^	10.1 ± 1.6 ^ab^	35.8 ± 1.1 ^cd^	9.5 ± 0.1 ^abcd^	45.3 ± 1.2 ^e^
Sample×Day	AX_HYD *	3	1	2.9 ± 0.2 ^abc^	8.6 ± 0.4 ^ab^	34.9 ± 0.5 ^cd^	9.5 ± 0.0 ^abcd^	44.4 ± 0.5 ^e^
Sample×Day	AX_HYD *	0	2	4.5 ± 0.4 ^cd^	8.4 ± 1.5 ^ab^	34.0 ± 0.9 ^bcd^	8.0 ± 0.1 ^abcd^	42.0 ± 0.7 ^bcde^
Sample×Day	AX_HYD *	1	2	4.4 ± 0.6 ^bcd^	8.8 ± 0.1 ^ab^	34.6 ± 1.1 ^cd^	10.0 ± 0.4 ^bcd^	44.7 ± 0.8 ^e^
Sample×Day	AX_HYD *	3	2	3.2 ± 0.4 ^abcd^	8.6 ± 0.6 ^ab^	33.8 ± 1.6 ^abcd^	10.6 ± 0.8 ^cd^	44.3 ± 0.9 ^e^
Sample×Day	AX_CR *	0	1	3.7 ± 0.6 ^abcd^	9.3 ± 0.2 ^ab^	29.1 ± 1.3 ^ab^	8.3 ± 0.3 ^abcd^	37.4 ± 1.0 ^ab^
Sample×Day	AX_CR *	1	1	3.2 ± 0.4 ^abcd^	8.5 ± 0.9 ^ab^	33.0 ± 1.3 ^abcd^	8.3 ± 0.3 ^abcd^	41.3 ± 1.0 ^abcde^
Sample×Day	AX_CR *	3	1	2.9 ± 0.5 ^abcd^	8.9 ± 0.3 ^ab^	33.9 ± 0.7 ^abcd^	9.1 ± 1.4 ^abcd^	43.0 ± 0.7 ^cde^
Sample×Day	AX_CR *	0	2	4.1 ± 0.0 ^abcd^	9.5 ± 0.1 ^ab^	28.9 ± 0.7 ^a^	7.4 ± 0.1 ^abc^	36.3 ± 0.6 ^a^
Sample×Day	AX_CR *	1	2	3.9 ± 0.3 ^abcd^	10.3 ± 0.3 ^ab^	32.8 ± 1.6 ^abcd^	8.7 ± 0.4 ^abcd^	41.5 ± 1.2 ^abcde^
Sample×Day	AX_CR *	3	2	2.7 ± 0.2 ^a^	9.1 ± 0.3 ^ab^	29.4 ± 1.1 ^ab^	11.0 ± 1.3 ^d^	40.3 ± 0.2 ^abcde^

* AX_NM—non-modified AX; AX_HYD—hydrolyzed AX; AX_CR—cross-linked AX. *** RDS—rapidly digestible starch; SDS—slowly digestible starch; DS—digestible starch; RS—resistant starch; TS—total starch. Mean values marked with the same letters in particular columns are not statistically significantly different at *p* < 0.05.

**Table 3:** Molecular properties of resistant starch extracted from the crumb of wheat bread with arabinoxylan (AX) preparations baked with sourdough or the postponed baking method on the day of baking.

	Level of	Technology **	Share [%]	M_w_ *** [g/mol]	M_n_ *** [g/mol]	Ð *** [-]
Technology	SD			18,860 ± 6298 ^a^	5544 ± 1063 ^a^	3.35 ± 0.47 ^a^
Technology	PB			28,401 ± 3208 ^b^	6671 ± 488 ^b^	4.25 ± 0.34 ^b^
Preparation	Control			21,505 ± 6120 ^a^	5916 ± 640 ^a^	3.58 ± 0.66 ^a^
Preparation	AX_NM *			26,768 ± 6884 ^b^	6768 ± 724 ^b^	3.91 ± 0.65 ^d^
Preparation	AX_HYD *			23,253 ± 7136 ^a^	5951 ± 918 ^a^	3.84 ± 0.72 ^c^
Preparation	AX_CR *			21,935 ± 7215 ^a^	5700 ± 1239 ^a^	3.77 ± 0.52 ^b^
Share [%]	0			21,505 ± 6120 ^a^	5916 ± 640 ^a^	3.58 ± 0.66 ^a^
Share [%]	1			22,233 ± 5856 ^a^	6040 ± 862 ^a^	3.65 ± 0.61 ^b^
Share [%]	2			25,737 ± 7987 ^b^	6239 ± 1239 ^a^	4.03 ± 0.57 ^c^
Sample×Technology	Control	SD	0	16,240 ± 919 ^a^	5398 ± 316 ^abc^	3.01 ± 0.01 ^a^
Sample×Technology	AX_NM *	SD	1	18,540 ± 524 ^ab^	6130 ± 187 ^bcd^	3.02 ± 0.01 ^a^
Sample×Technology	AX_NM *	SD	2	33,390 ± 850 ^e^	7600 ± 221 ^e^	4.39 ± 0.02 ^i^
Sample×Technology	AX_HYD *	SD	1	17,470 ± 494 ^a^	5775 ± 170 ^abc^	3.03 ± 0.00 ^a^
Sample×Technology	AX_HYD *	SD	2	15,820 ± 1342 ^a^	4695 ± 418 ^a^	3.37 ± 0.01 ^c^
Sample×Technology	AX_CR *	SD	1	15,770 ± 245 ^a^	4540 ± 85 ^a^	3.47 ± 0.01 ^d^
Sample×Technology	AX_CR *	SD	2	14,790 ± 418 ^a^	4670 ± 160 ^a^	3.17 ± 0.02 ^b^
Sample×Technology	Control	PB	0	26,770 ± 795 ^cd^	6435 ± 226 ^bcde^	4.16 ± 0.02 ^g^
Sample×Technology	AX_NM *	PB	1	22,640 ± 480 ^bc^	6190 ± 145 ^bcd^	3.66 ± 0.01 ^e^
Sample×Technology	AX_NM *	PB	2	32,500 ± 2758 ^e^	7150 ± 633 ^de^	4.55 ± 0.02 ^j^
Sample×Technology	AX_HYD *	PB	1	30,110 ± 1277 ^de^	6395 ± 278 ^bcde^	4.71 ± 0.01 ^k^
Sample×Technology	AX_HYD *	PB	2	29,610 ± 754 ^de^	6940 ± 218 ^cde^	4.27 ± 0.03 ^h^
Sample×Technology	AX_CR *	PB	1	28,870 ± 2450 ^de^	7210 ± 559 ^de^	4.00 ± 0.03 ^f^
Sample×Technology	AX_CR *	PB	2	28,310 ± 1601 ^de^	6380 ± 334 ^bcde^	4.44 ± 0.02 ^i^

* AX_NM—non-modified AX; AX_HYD—hydrolyzed AX; AX_CR—cross-linked AX. ** SD—sourdough baking method; PB—postponed baking method. *** M_w_—weight-average molar mass; M_n_—number-average molar mass; Ð—dispersity (M_w_/M_n_). Mean values marked with the same letters in particular columns are not statistically significantly different at *p* < 0.05.

**Table 4:** Molecular properties of resistant starch extracted from the crumb of wheat bread with arabinoxylan (AX) preparation baked with sourdough on the day of baking and after 1 and 3 days of storage.

	Level of	Day of Storage	Share [%]	M_w_ ** [g/mol]	M_n_ ** [g/mol]	Ð [-]
Preparation	Control			22,963 ± 5356 ^a^	5296 ± 302 ^ab^	4.35 ± 1.04 ^c^
Preparation	AX_NM *			26,377 ± 5206 ^b^	6370 ± 678 ^c^	4.14 ± 0.72 ^a^
Preparation	AX_HYD *			23,232 ± 5065 ^a^	5518 ± 462 ^b^	4.19 ± 0.75 ^b^
Preparation	AX_CR *			23,765 ± 6739 ^a^	5128 ± 445 ^a^	4.58 ± 1.04 ^d^
Share [%]	0			22,963 ± 5356 ^a^	5296 ± 302 ^a^	4.35 ± 1.04 ^b^
Share [%]	1			23,004 ± 4451 ^a^	5650 ± 614 ^b^	4.09 ± 0.77 ^a^
Share [%]	2			25,911 ± 6575 ^b^	5694 ± 870 ^b^	4.52 ± 0.89 ^c^
Day	0			18,860 ± 6293 ^a^	5544 ± 1063 ^a^	3.35 ± 0.47 ^a^
Day	1			26,880 ± 2880 ^b^	5606 ± 364 ^a^	4.81 ± 0.60 ^c^
Day	3			26,993 ± 2341 ^b^	5704 ± 545 ^a^	4.76 ± 0.53 ^b^
Sample×Day	Control	0	0	16,240 ± 919 ^a^	5398 ± 316 ^abc^	3.01 ± 0.01 ^a^
Sample×Day	Control	1	0	25,930 ± 1100 ^cde^	5100 ± 230 ^abc^	5.08 ± 0.01 ^l^
Sample×Day	Control	3	0	26,720 ± 2267 ^cdef^	5390 ± 435 ^abc^	4.96 ± 0.02 ^k^
Sample×Day	AX_NM *	0	1	18,540 ± 524 ^ab^	6130 ± 187 ^cd^	3.02 ± 0.01 ^a^
Sample×Day	AX_NM *	1	1	28,060 ± 595 ^cdef^	5875 ± 146 ^cd^	4.78 ± 0.02 ^j^
Sample×Day	AX_NM *	3	1	24,660 ± 1395 ^cd^	6670 ± 391 ^de^	3.70 ± 0.01 ^e^
Sample×Day	AX_NM *	0	2	33,390 ± 850 ^g^	7600 ± 221 ^e^	4.39 ± 0.02 ^g^
Sample×Day	AX_NM *	1	2	23,070 ± 1958 ^bc^	5915 ± 522 ^cd^	3.90 ± 0.01 ^f^
Sample×Day	AX_NM *	3	2	30,540 ± 1296 ^efg^	6030 ± 270 ^cd^	5.06 ± 0.01 ^l^
Sample×Day	AX_HYD *	0	1	17,470 ± 494 ^a^	5775 ± 270 ^bcd^	3.03 ± 0.00 ^a^
Sample×Day	AX_HYD *	1	1	26,470 ± 2246 ^cde^	5745 ± 426 ^bcd^	4.61 ± 0.05 ^hi^
Sample×Day	AX_HYD *	3	1	24,960 ± 635 ^cd^	5370 ± 151 ^abc^	4.65 ± 0.01 ^i^
Sample×Day	AX_HYD *	0	2	15,820 ± 1342 ^a^	4695 ± 418 ^ab^	3.37 ± 0.01 ^c^
Sample×Day	AX_HYD *	1	2	28,250 ± 599 ^defg^	5695 ± 142 ^abcd^	4.96 ± 0.02 ^k^
Sample×Day	AX_HYD *	3	2	26,420 ± 673 ^cde^	5830 ± 176 ^bcd^	4.53 ± 0.02 ^h^
Sample×Day	AX_CR *	0	1	15,770 ± 245 ^a^	4540 ± 85 ^a^	3.47 ± 0.01 ^d^
Sample×Day	AX_CR *	1	1	24,700 ± 699 ^cd^	5565 ± 178 ^abcd^	4.44 ± 0.02 ^g^
Sample×Day	AX_CR *	3	1	26,410 ± 2241 ^cde^	5180 ± 393 ^abc^	5.10 ± 0.05 ^l^
Sample×Day	AX_CR *	0	2	14,790 ± 418 ^a^	4670 ± 160 ^ab^	3.17 ± 0.02 ^b^
Sample×Day	AX_CR *	1	2	31,680 ± 1792 ^fg^	5350 ± 330 ^abc^	5.92 ± 0.03 ^n^
Sample×Day	AX_CR *	3	2	29,240 ± 827 ^defg^	5460 ± 182 ^abc^	5.36 ± 0.03 ^m^

* AX_NM—non-modified AX; AX_HYD—hydrolyzed AX; AX_CR—cross-linked AX. ** M_w_—weight-average molar mass; M_n_—number-average molar mass; Ð—dispersity (M_w_/M_n_). Mean values marked with the same letters in particular columns are not statistically significantly different at *p* < 0.05.

**Table 5:** Results of amylographic analysis of the crumb of wheat bread with arabinoxylan (AX) preparations baked with sourdough and using the postponed baking method on the day of baking.

	Level of	Technology **	Share [%]	Initial Viscosity [BU]	PT *** [°C]	Maximum Viscosity [BU]	Viscosity at 50 °C [BU]
Technology	SD			15 ± 2 ^a^	89.3 ± 1.7 ^b^	76 ± 12 ^a^	139 ± 23 ^a^
Technology	PB			21 ± 4 ^b^	88.2 ± 1.7 ^a^	270 ± 51 ^b^	386 ± 83 ^b^
Preparation	Control			13 ± 2 ^a^	90.5 ± 0.6 ^b^	183 ± 104 ^b^	273 ± 115 ^b^
Preparation	AX_NM *			20 ± 6 ^b^	89.3 ± 1.8 ^ab^	187 ± 120 ^b^	283 ± 148 ^c^
Preparation	AX_HYD *			18 ± 3 ^b^	88.1 ± 1.4 ^a^	131 ± 73 ^a^	194 ± 90 ^a^
Preparation	AX_CR *			18 ± 3 ^b^	88.2 ± 1.8 ^a^	196 ± 125 ^c^	306 ± 176 ^d^
Share [%]	0			13 ± 2 ^a^	90.5 ± 0.6 ^b^	183 ± 104 ^b^	273 ± 115 ^c^
Share [%]	1			17 ± 3 ^b^	89.4 ± 1.4 ^b^	173 ± 105 ^a^	258 ± 133 ^a^
Share [%]	2			20 ± 5 ^c^	87.6 ± 1.6 ^a^	170 ± 115 ^a^	264 ± 161 ^b^
Sample×Technology	Control	SD	0	11 ± 1 ^a^	90.7 ± 0.9 ^de^	93 ± 7 ^b^	173 ± 3 ^d^
Sample×Technology	AX_NM *	SD	1	13 ± 1 ^ab^	91.7 ± 0.5 ^e^	72 ± 10 ^ab^	144 ± 4 ^c^
Sample×Technology	AX_NM *	SD	2	17 ± 1 ^bcd^	89.7 ± 1.3 ^bcde^	78 ± 5 ^b^	145 ± 2 ^c^
Sample×Technology	AX_HYD *	SD	1	16 ± 1 ^abc^	89.4 ± 0.8 ^bcde^	77 ± 6 ^b^	129 ± 1 ^b^
Sample×Technology	AX_HYD *	SD	2	17 ± 1 ^bcd^	86.8 ± 1.1 ^ab^	53 ± 1 ^a^	95 ± 1 ^a^
Sample×Technology	AX_CR *	SD	1	16 ± 1 ^abc^	88.2 ± 0.9 ^abcd^	79 ± 4 ^b^	138 ± 1 ^bc^
Sample×Technology	AX_CR *	SD	2	15 ± 1 ^abc^	88.8 ± 0.9 ^abcde^	79 ± 6 ^b^	150 ± 1 ^c^
Sample×Technology	Control	PB	0	14 ± 0 ^ab^	90.3 ± 0.4 ^cde^	272 ± 8 ^e^	373 ± 5 ^g^
Sample×Technology	AX_NM *	PB	1	21 ± 1 ^de^	87.8 ± 0.4 ^abcd^	296 ± 1 ^f^	411 ± 4 ^h^
Sample×Technology	AX_NM *	PB	2	28 ± 1 ^f^	87.9 ± 1.3 ^abcd^	302 ± 2 ^f^	432 ± 5 ^i^
Sample×Technology	AX_HYD *	PB	1	20 ± 1 ^cde^	89.1 ± 0.4 ^bcde^	216 ± 3 ^d^	298 ± 4 ^f^
Sample×Technology	AX_HYD *	PB	2	21 ± 3 ^de^	87.0 ± 0.1 ^abc^	178 ± 7 ^c^	254 ± 1 ^e^
Sample×Technology	AX_CR *	PB	1	21 ± 1 ^de^	90.0 ± 0.8 ^bcde^	297 ± 6 ^f^	428 ± 5 ^i^
Sample×Technology	AX_CR *	PB	2	22 ± 0 ^e^	85.7 ± 0.8 ^a^	328 ± 4 ^g^	508 ± 5 ^j^

* AX_NM—non-modified AX; AX_HYD—hydrolyzed AX; AX_CR—cross-linked AX. ** SD—sourdough baking method; PB—postponed baking method. *** PT—pasting temperature. Mean values marked with the same letters in particular columns are not statistically significantly different at *p* < 0.05.

**Table 6:** Results of amylographic analysis of the crumb of wheat bread with arabinoxylan (AX) preparations baked with sourdough on the day of baking and after 1 and 3 days of storage.

	Level of	Day of Storage	Share [%]	Initial Viscosity [BU]	PT ** [°C]	Maximum Viscosity [BU]	Viscosity at 50 °C [BU]
Preparation	Control			8 ± 3 ^a^	88.2 ± 2.1 ^bc^	85 ± 12 ^a^	152 ± 22 ^d^
Preparation	AX_NM *			12 ± 3 ^c^	88.7 ± 2.5 ^c^	74 ± 9 ^a^	139 ± 12 ^b^
Preparation	AX_HYD *			10 ± 5 ^b^	86.3 ± 2.6 ^a^	57 ± 12 ^a^	102 ± 17 ^a^
Preparation	AX_CR *			12 ± 3 ^c^	87.4 ± 1.5 ^b^	80 ± 9 ^a^	149 ± 17 ^c^
Share [%]	0			8 ± 3 ^a^	88.2 ± 2.1 ^b^	85 ± 12 ^a^	152 ± 22 ^b^
Share [%]	1			11 ± 3 ^b^	88.1 ± 2.3 ^b^	74 ± 10 ^a^	134 ± 17 ^b^
Share [%]	2			12 ± 4 ^c^	86.8 ± 2.3 ^a^	67 ± 17 ^a^	126 ± 32 ^a^
Day	0			15 ± 2 ^c^	89.3 ± 1.7 ^c^	76 ± 12 ^a^	139 ± 23 ^c^
Day	1			10 ± 2 ^b^	87.2 ± 1.5 ^b^	73 ± 17 ^a^	133 ± 27 ^b^
Day	3			8 ± 2 ^a^	86.2 ± 2.6 ^a^	69 ± 15 ^a^	128 ± 29 ^a^
Sample×Day	Control	0	0	11 ± 1 ^fghi^	90.7 ± 0.9 ^gh^	93 ± 7 ^e^	173 ± 3 ^m^
Sample×Day	Control	1	0	7 ± 1 ^abc^	86.9 ± 0.8 ^bcdef^	91 ± 7 ^e^	159 ± 3 ^kl^
Sample×Day	Control	3	0	6 ± 1 ^a^	86.9 ± 0.8 ^bcdef^	72 ± 6 ^bcde^	125 ± 1 ^ef^
Sample×Day	AX_NM *	0	1	13 ± 1 ^ijk^	91.7 ± 0.5 ^h^	72 ± 10 ^bcde^	144 ± 4 ^hij^
Sample×Day	AX_NM *	1	1	13 ± 1 ^hijk^	86.3 ± 0.9 ^bcde^	83 ± 8 ^cde^	148 ± 1 ^ij^
Sample×Day	AX_NM *	3	1	10 ± 1 ^cdefgh^	90.3 ± 0.4 ^fgh^	70 ± 6 ^bcd^	132 ± 3 ^fg^
Sample×Day	AX_NM *	0	2	17 ± 1 ^l^	89.7 ± 1.3 ^efgh^	78 ± 5 ^cde^	145 ± 2 ^hij^
Sample×Day	AX_NM *	1	2	12 ± 1 ^fghi^	89.4 ± 0.8 ^efgh^	63 ± 4 ^abc^	118 ± 1 ^de^
Sample×Day	AX_NM *	3	2	9 ± 1 ^abcdef^	85.0 ± 0.8 ^abc^	82 ± 4 ^cde^	151 ± 4 ^jk^
Sample×Day	AX_HYD *	0	1	16 ± 1 ^kl^	89.4 ± 0.8 ^efgh^	77 ± 6 ^cde^	129 ± 1 ^f^
Sample×Day	AX_HYD *	1	1	8 ± 1 ^abcde^	88.8 ± 0.9 ^defgh^	62 ± 4 ^abc^	109 ± 1 ^c^
Sample×Day	AX_HYD *	3	1	6 ± 0 ^ab^	84.4 ± 0.8 ^ab^	63 ± 1 ^abc^	112 ± 2 ^cd^
Sample×Day	AX_HYD *	0	2	17 ± 1 ^l^	86.8 ± 1.1 ^bcde^	53 ± 1 ^ab^	95 ± 1 ^b^
Sample×Day	AX_HYD *	1	2	9 ± 0 ^bcdefg^	86.3 ± 0.9 ^bcde^	46 ± 1 ^a^	89 ± 1 ^b^
Sample×Day	AX_HYD *	3	2	7 ± 0 ^abcd^	82.5 ± 0.8 ^a^	42 ± 1 ^a^	78 ± 1 ^a^
Sample×Day	AX_CR *	0	1	16 ± 1 ^kl^	88.2 ± 0.9 ^cdefgh^	79 ± 4 ^cde^	138 ± 1 ^gh^
Sample×Day	AX_CR *	1	1	11 ± 1 ^efghi^	85.7 ± 0.9 ^abcd^	91 ± 6 ^de^	166 ± 1 ^lm^
Sample×Day	AX_CR *	3	1	10 ± 0 ^defghi^	88.8 ± 0.9 ^defgh^	68 ± 6 ^bc^	125 ± 3 ^ef^
Sample×Day	AX_CR *	0	2	15 ± 1 ^jkl^	88.8 ± 0.9 ^defgh^	79 ± 6 ^cde^	150 ± 1 ^j^
Sample×Day	AX_CR *	1	2	12 ± 1 ^fghi^	87.5 ± 0.8 ^bcdefg^	74 ± 5 ^bcde^	140 ± 3 ^ghi^
Sample×Day	AX_CR *	3	2	12 ± 0 ^ghij^	85.7 ± 0.9 ^abcd^	90 ± 4 ^de^	172 ± 3 ^m^

* AX_NM—non-modified AX; AX_HYD—hydrolyzed AX; AX_CR—cross-linked AX. ** PT—pasting temperature. Mean values marked with the same letters in particular columns are not statistically significantly different at *p* < 0.05.

## Data Availability

Data are contained within the article.

## Supplementary Materials

The following supporting information can be downloaded at: https://www.mdpi.com/article/10.3390/molecules30081722/s1, Figure S1. Molar mass distribution profiles of resistant starch extracted from the crumb of wheat bread baked: (a) with non-modified arabinoxylans (AX_NM); (b) with hydrolyzed AX (AX_HYD); (c) cross-linked AX (AX_CR) by the postponed baking method (-●- without AX, -□- with 1% AX, -∆- with 2% AX); Figure S2. Molar mass distribution profiles of resistant starch extracted from the crumb: (a) with non-modified arabinoxylans (AX_NM); (b) with hydrolyzed AX (AX_HYD); (c) cross-linked AX (AX_CR) of sourdough wheat bread (-●- without AX, -□- with 1% AX, -∆- with 2% AX); Figure S3. Molar mass distribution profiles of resistant starch extracted from the crumb: (a) resistant starch standard; (b) control without share of AX; (c) with 1% share of non-modified arabinoxylans (AX_NM); (d) with 2% share of non-modified arabinoxylans (AX_NM); (e) with 1% share of hydrolyzed arabinoxylans (AX_HYD); (f) with 2% share of hydrolyzed arabinoxylans (AX_HYD); (g) with 1% share of cross-linked arabinoxylans (AX_CR); (h) with 2% share of cross-linked arabinoxylans (AX_CR) of sourdough wheat bread (-●- on the day of baking, -□- during 1 day of storage, -∆- during 3 days of storage); Figure S4. Amylographic characterization of suspensions prepared from lyophilized crumb: (a) with non-modified arabinoxylans (AX_NM); (b) with hydrolyzed AX (AX_HYD); (c) cross-linked AX (AX_CR) of wheat breads baked by postponed baking method (-●- without AX (control), -∆- with 1% AX, -○- with 2% AX); Figure S5. Amylographic characterization of suspensions prepared from lyophilized crumb: (a) with non-modified arabinoxylans (AX_NM); (b) with hydrolyzed AX (AX_HYD); (c) cross-linked AX (AX_CR) of sourdough wheat breads (-●- without AX (control), -∆- with 1% AX, -○- with 2% AX); Figure S6. Amylographic characterization of suspensions prepared from lyophilized crumb: (a) control without share of AX; (b) with 1% share of non-modified arabinoxylans (AX_NM); (c) with 2% share of non-modified arabinoxylans (AX_NM); (d) with 1% share of hydrolyzed arabinoxylans (AX_HYD); (e) with 2% share of hydrolyzed arabinoxylans (AX_HYD); (f) with 1% share of cross-linked arabinoxylans (AX_CR); (g) with 2% share of cross-linked arabinoxylans (AX_CR)) of sourdough wheat breads (-●- on the day of baking, -∆- during 1 day of storage, -○- during 3 days of storage); Table S1. Basic characteristics of arabinoxylan (AX) preparations [48,49]; Table S2. The influence of the addition of arabinoxylans with different molar mass on the properties of bread baked by the postponed and sourdough methods, obtained on the day of baking and during storage [48,49].
