# Supplementary material for: Characteristics of In-Vitro Starch Digestibility in Wheat Bread with Arabinoxylans, Baked Using Sourdough or Postponed Baking Methods
Source: Molecules. 2025 Apr 11;30(8):1722. doi: 10.3390/molecules30081722 (PMC12029739; doi:10.3390/molecules30081722)
Supplement: Supplementary file 1 [file molecules-30-01722-s001.zip › molecules-3460703-supplementary.pdf]

a)

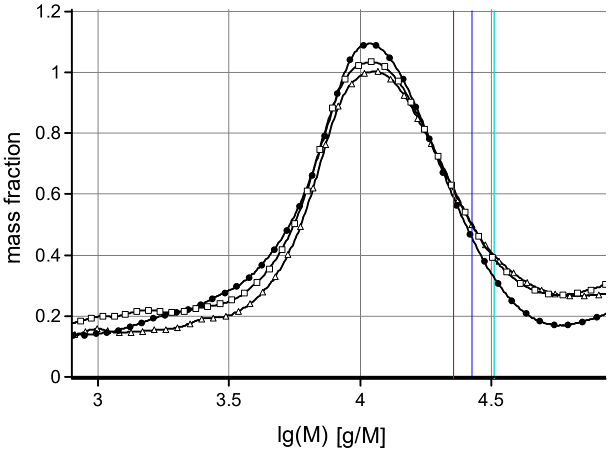

b)

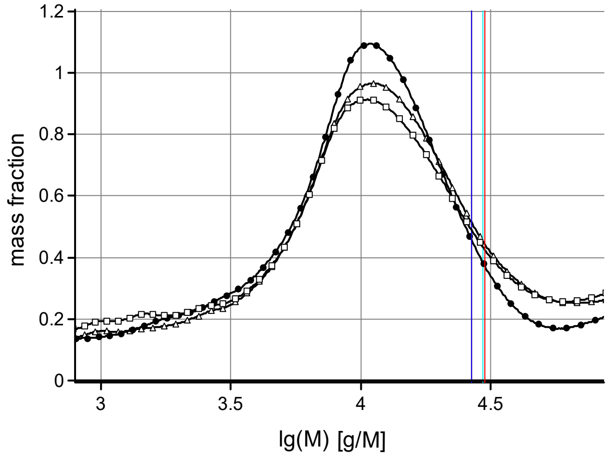

c)

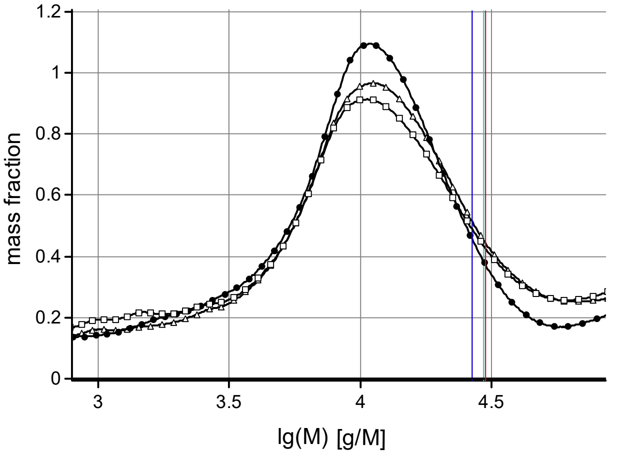

Supplementary Figure S1. Molar mass distribution profiles of resistant starch extracted from the crumb of wheat bread baked: a) with non-modified arabinoxylans (AX_NM); b) with hydrolyzed AX (AX_HYD); c) cross-linked AX (AX_CR) by the postponed baking method (-●- without AX, -□- with 1% AX, -Δ- with 2% AX).

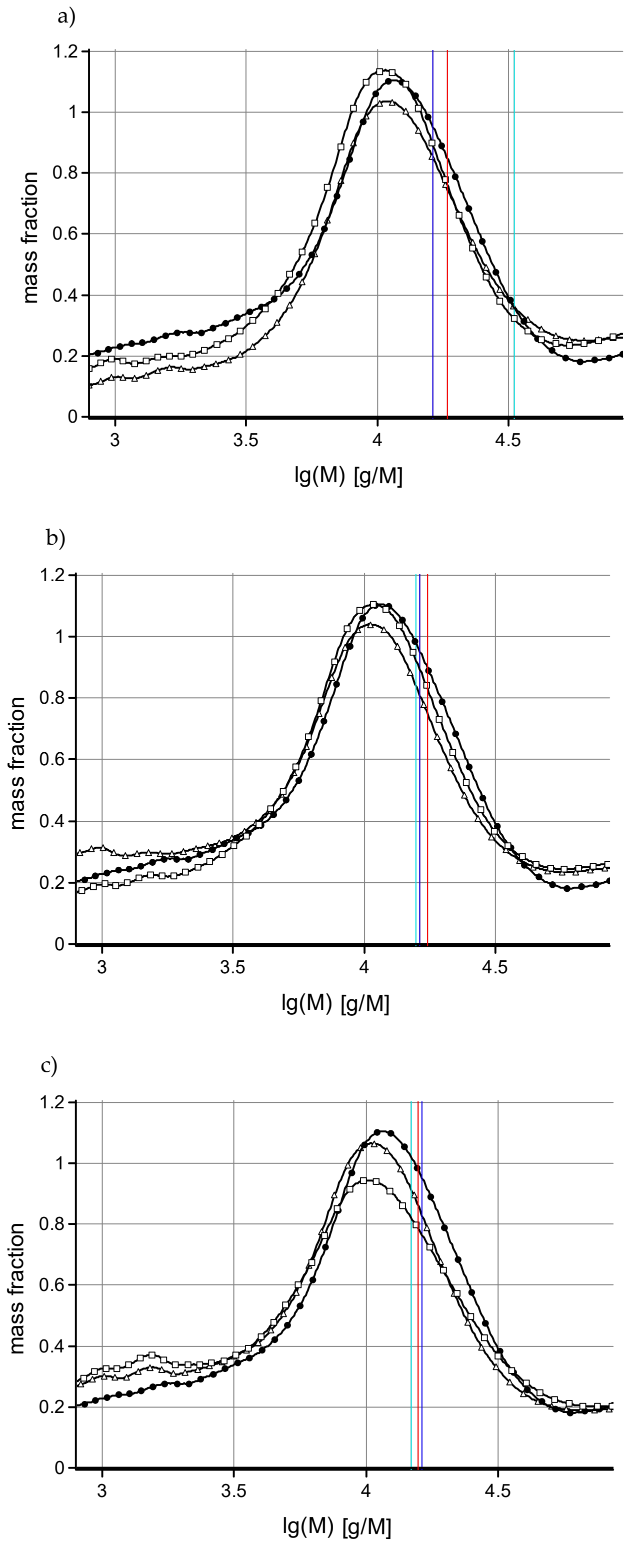

Supplementary Figure S2. Molar mass distribution profiles of resistant starch extracted from the crumb: a) with non-modified arabinoxylans (AX_NM); b) with **h**ydrolyzed AX (AX_HYD); c) cross-linked AX (AX_CR) of sourdough wheat bread (-●- without AX, -□- with 1% AX, -Δ- with 2% AX).

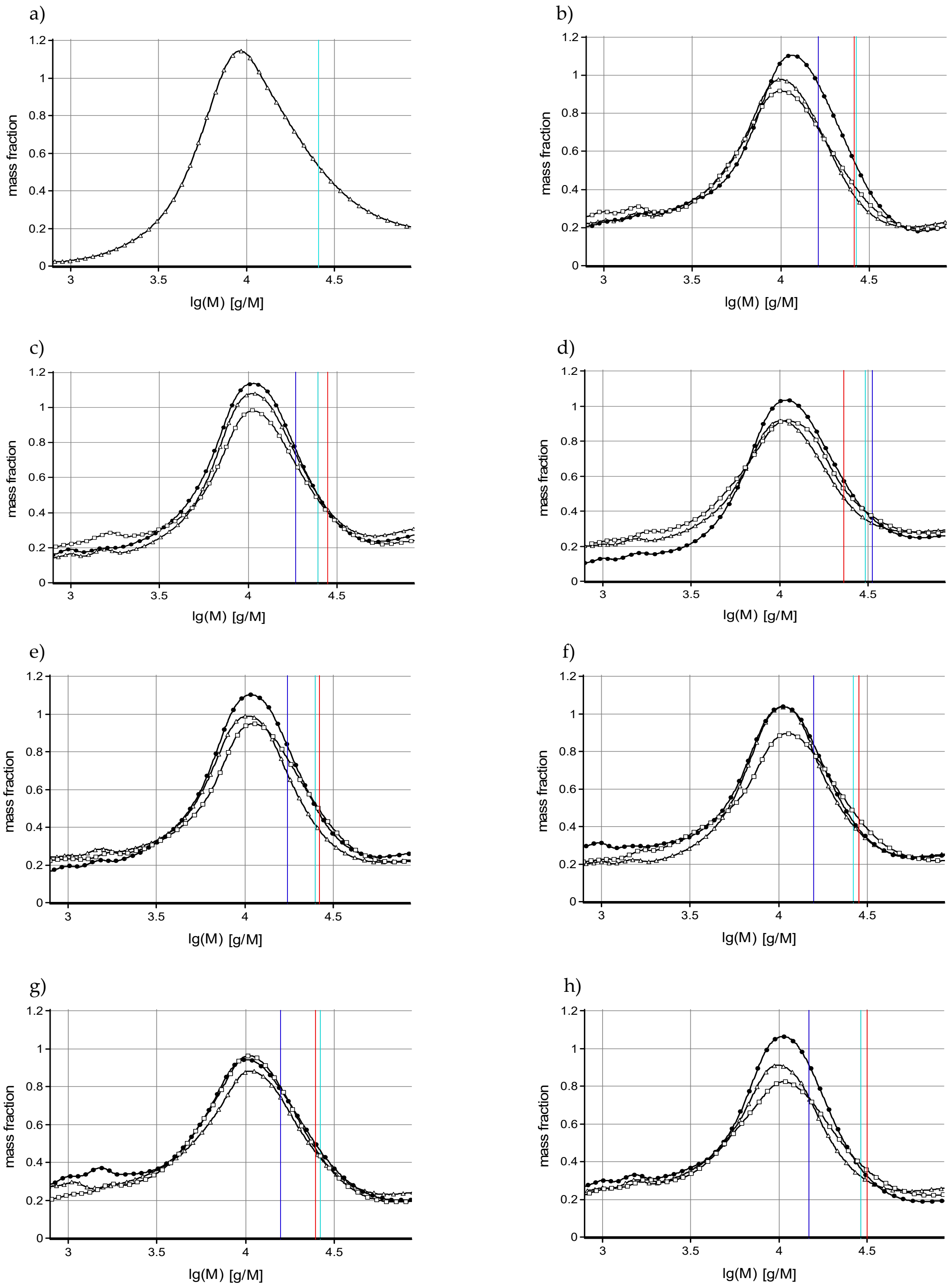

Supplementary Figure S3. Molar mass distribution profiles of resistant starch extracted from the crumb: a) resistant starch standard; b) control without share of AX; c) with 1% share of non-modified arabinoxylans (AX_NM); d) with 2% share of non-modified arabinoxylans (AX_NM); e) with 1% share of hydrolyzed arabinoxylans (AX_HYD); f) with 2% share of hydrolyzed arabinoxylans (AX_HYD); g) with 1% share of cross-linked arabinoxylans (AX_CR); h) with 2% share of cross-linked arabinoxylans (AX_CR) of sourdough wheat bread (-●- on the day of baking, -□- during 1 day of storage, -Δ- during 3 days of storage).

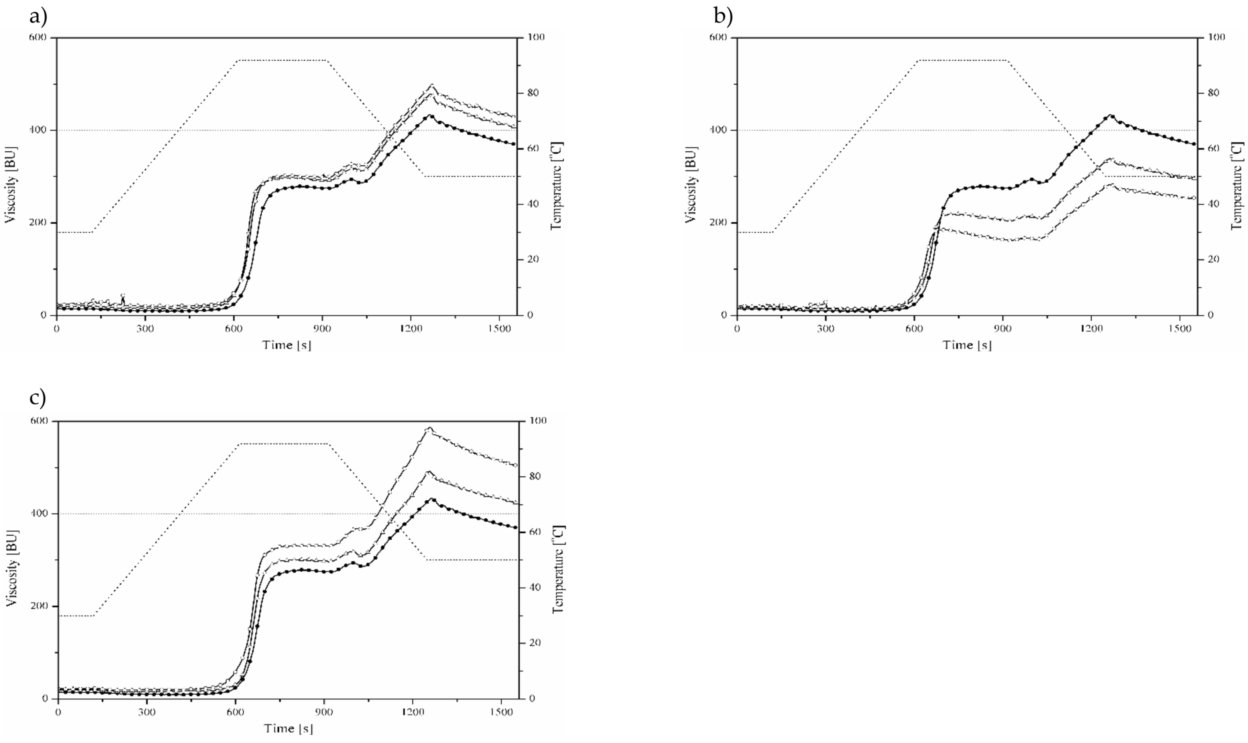

Supplementary Figure S4. Amylographic characterization of suspensions prepared from lyophilized crumb: a) with non-modified arabinoxylans (AX_NM); b) with hydrolyzed AX (AX_HYD); c) cross-linked AX (AX_CR) of wheat breads baked by postponed baking method (-●- without AX (control), -∆- with 1% AX, -○- with 2% AX).

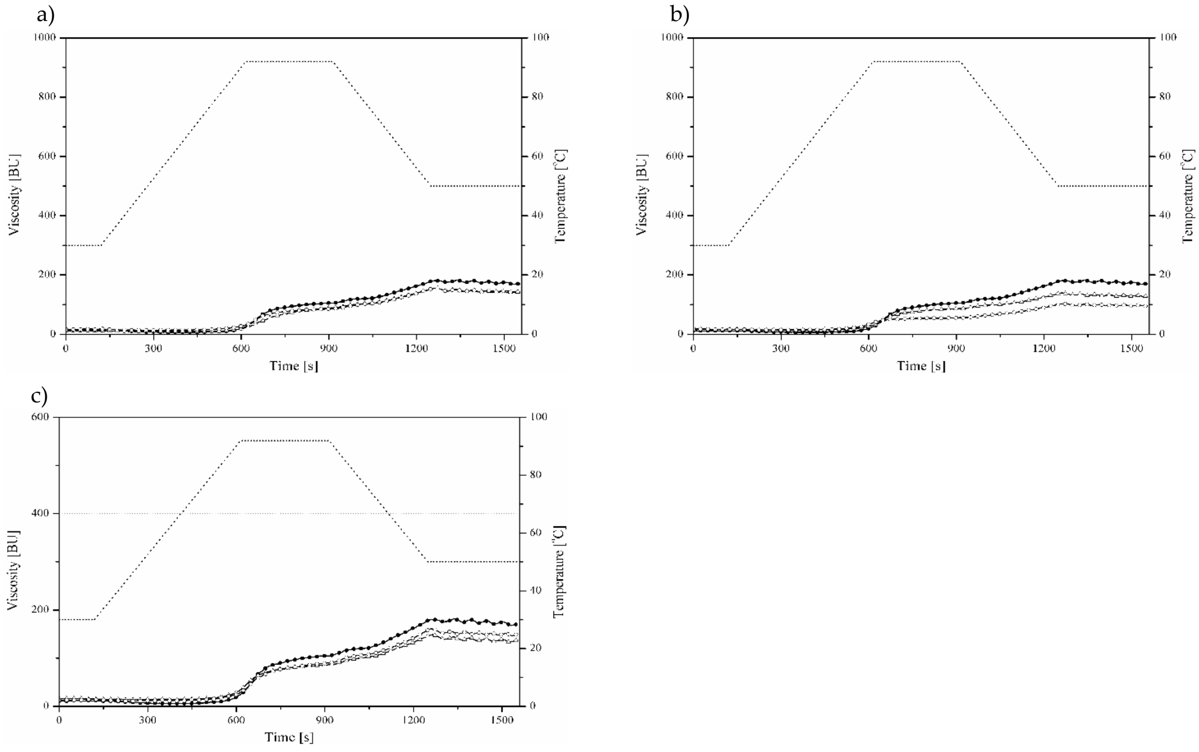

Supplementary Figure S5. Amylographic characterization of suspensions prepared from lyophilized crumb: a) with non-modified arabinoxylans (AX_NM); b) with hydrolyzed AX (AX_HYD); c) cross-linked AX (AX_CR) of sourdough wheat breads (-●- without AX (control), -∆- with 1% AX, -○- with 2% AX).

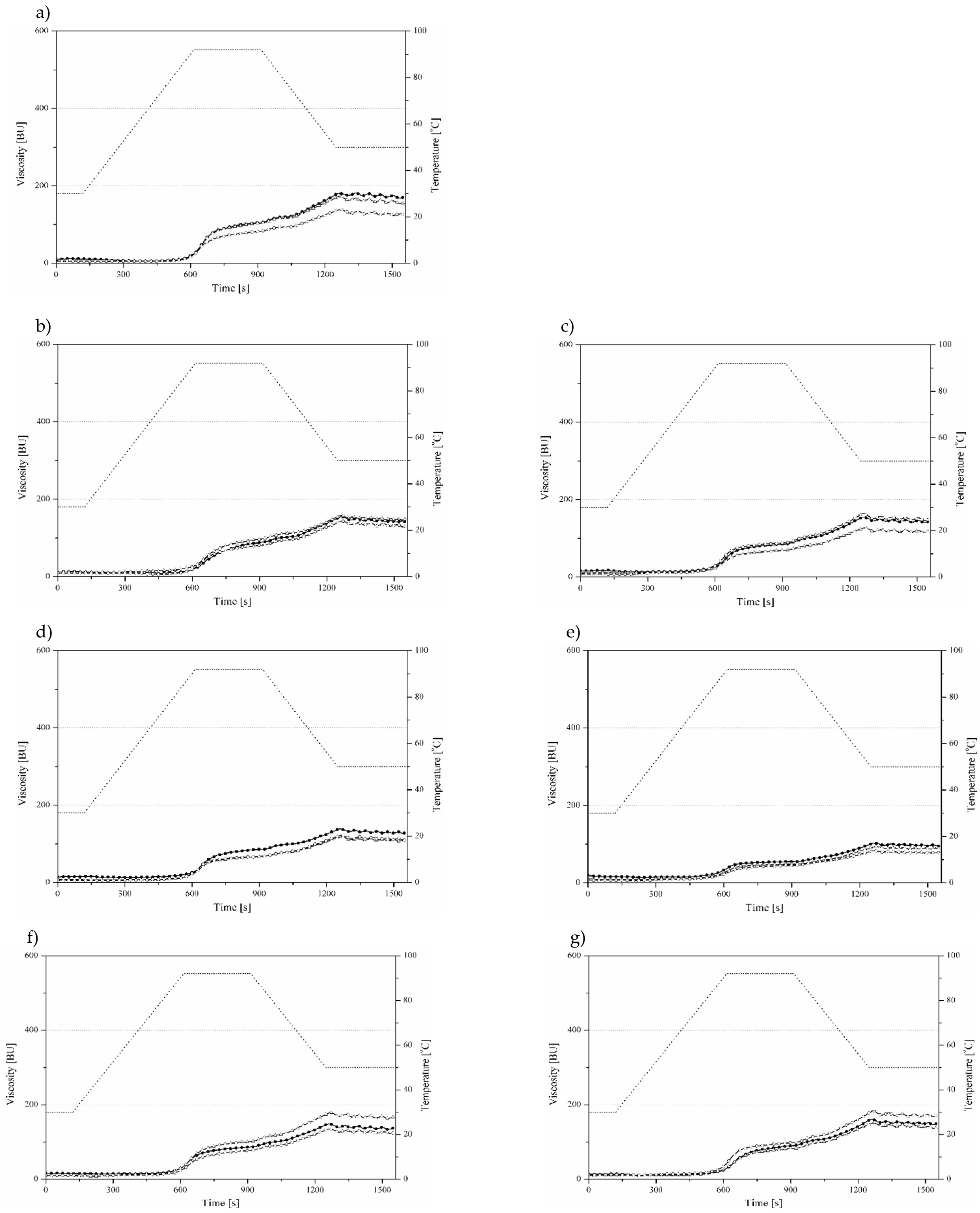

Supplementary Figure S6. Amylographic characterization of suspensions prepared from lyophilized crumb: a) control without share of AX; b) with 1% share of non-modified arabinoxylans (AX_NM); c) with 2% share of non-modified arabinoxylans (AX_NM); d) with 1% share of hydrolyzed arabinoxylans (AX_HYD); e) with 2% share of hydrolyzed arabinoxylans (AX_HYD); f) with 1% share of cross-linked arabinoxylans (AX_CR); g) with 2% share of cross-linked arabinoxylans (AX_CR)) of sourdough wheat breads (-●- on the day of baking, -∆- during 1 day of storage, -○- during 3 days of storage).

Supplementary Table S1. Basic characteristics of arabinoxylan (AX) preparations

Preparation	AX content [%]	Molar mass of AX [g/mol]	Reference
Preparations used in postponed baking *			
AX_NM	76.0	413,800	[49]
AX_HYD	75.0	192,320	
AX_CR	75.7	535,630	
Preparations used in sourdough baking **			
AX_NM	78.2	446,330	[48]
AX_HYD	77.1	190,440	
AX_CR	76.6	574,710	

* detailed characteristics is presented in publication Bieniek and Buksa 2024 [49].

** detailed characteristics is presented in publication Bieniek and Buksa 2024 [48].

Supplementary Table S2. The influence of the addition of arabinoxylans with different molar mass on the properties of bread baked by the postponed and sourdough methods, obtained on the day of baking and during storage.

Sample *	Share [%]	Technology **	Day of storage	WBC *** [%]	SBV *** [cm³/100 g of flour]	Crumb Hardness [N]	Crumb Moisture—Central Part [%]
Sourdough breads ****							
Control	0	SD	0	56.2 ± 0.2	316.7 ± 3.6	9.7 ± 0.4	41.7 ± 0.3
	0	SD	1	-	-	17.5 ± 0.1	41.2 ± 0.1
	0	SD	3	-	-	28.9 ± 0.2	40.1 ± 0.0
AX_NM	1	SD	0	60.6 ± 0.2	331.2 ± 4.0	9.7 ± 0.4	43.2 ± 0.5
	1	SD	1	-	-	17.8 ± 1.7	43.1 ± 0.4
	1	SD	3	-	-	31.2 ± 2.2	40.9 ± 0.4
AX_NM	2	SD	0	64.3 ± 0.2	379.9 ± 3.4	7.2 ± 0.3	43.6 ± 0.2
	2	SD	1	-	-	11.8 ± 0.1	43.5 ± 0.2
	2	SD	3	-	-	21.8 ± 0.8	42.9 ± 0.5
AX_HYD	1	SD	0	59.4 ± 0.5	367.3 ± 4.0	6.7 ± 0.3	42.8 ± 0.2
	1	SD	1	-	-	12.5 ± 0.1	42.2 ± 0.2
	1	SD	3	-	-	25.4 ± 2.3	40.1 ± 0.0
AX_HYD	2	SD	0	61.2 ± 0.2	391.6 ± 4.0	6.5 ± 0.4	43.4 ± 0.5
	2	SD	1	-	-	11.8 ± 1.4	42.4 ± 0.2
	2	SD	3	-	-	22.7 ± 1.2	41.3 ± 0.1
AX_CR	1	SD	0	63.0 ± 0.3	345.7 ± 1.4	8.8 ± 0.3	43.6 ± 0.4
	1	SD	1	-	-	14.3 ± 1.1	43.5 ± 0.5
	1	SD	3	-	-	28.0 ± 0.7	41.8 ± 0.4
AX_CR	2	SD	0	66.0 ± 0.4	347.2 ± 2.8	8.5 ± 0.4	44.3 ± 0.3
	2	SD	1	-	-	13.5 ± 0.1	44.1 ± 0.1
	2	SD	3	-	-	23.2 ± 1.4	43.2 ± 0.5
Postponed breads *****							
Control	0	PB	0	57.6 ± 0.3	397.9 ± 3.9	7.6 ± 0.3	43.7 ± 0.03
AX_NM	1	PB	0	61.0 ± 0.2	405.2 ± 2.7	6.5 ± 0.2	42.9 ± 0.6
AX_NM	2	PB	0	65.1 ± 0.1	376.8 ± 2.8	8.6 ± 0.3	44.3 ± 0.4
AX_HYD	1	PB	0	60.2 ± 0.4	432.0 ± 2.7	6.0 ± 0.2	42.3 ± 0.2
AX_HYD	2	PB	0	62.1 ± 0.2	414.5 ± 4.1	5.9 ± 0.3	43.5 ± 0.4
AX_CR	1	PB	0	63.4 ± 0.1	408.5 ± 0.0	6.8 ± 0.1	43.2 ± 0.8
AX_CR	2	PB	0	66.4 ± 0.2	406.3 ± 1.4	7.2 ± 0.1	45.03 ± 0.2

* Control- without share of AX; AX_NM-non-modified AX; AX_HYD-hydrolyzed AX; AX_CR-cross-linked AX. ** SD-sourdough, PB-postponed baking; *** WBC—water binding capacity, SBV—Specific bread volume. **** detailed characteristics is presented in publication Bieniek and Buksa 2024 [48]. ***** detailed characteristics is presented in publication Bieniek and Buksa 2024 [49]. Mean values marked with the same letters in particular rows are not statistically significantly different at $p < 0.05$.

Supplementary information - Modification of AX

1. Modification of Isolated Rye Arabinoxylans Using Cross-Linking

A total of 15 g of frozen precipitate was thawed and dissolved in 40 mL of deionized water with intense stirring at 50 °C for 6 h. The solution was then cooled to 25 °C, and hydrogen peroxide (1 μg/g AXs) and peroxidase (5 U/g AXs) were added and reacted for 15 min. The process was stopped by flooding the solution with a 4-volume solution of ethanol and acetone (1:1). The AX precipitate was centrifuged and washed twice with ethanol/acetone and twice with acetone only to remove all water from the sample. After the last centrifugation, the precipitate of AXs was dried at 50 °C for 2 h. The resulting preparation of cross-linked AX was denoted as AX_CR.

2. Modification of Isolated Rye Arabinoxylans Using Partial Enzymatic Hydrolysis

A total of 15 g of frozen precipitate was thawed and dissolved in 40 mL of deionized water with intense stirring at 50 °C for 6 h. After cooling the solution to 30 °C, xylanase (endo-β-1,4-xylanase) of Thermomyces lanuginosus (Merck Life Science Sp.z.o.o., an affiliate of Merck KGaA, Darmstadt, Germany) was added at 375 FXU/g AX and incubated at 37 °C for 30 min. After boiling, the sample was centrifuged, and the resulting supernatant was transferred to a 4-fold volume of ethanol/acetone solution (1:1). The precipitated AXs were centrifuged and washed twice with ethanol/acetone and twice with acetone only. After the last centrifugation, the precipitate of AXs was dried at 50 °C for 2 h. The resulting preparation of partly hydrolyzed AX was denoted as AX_HYD.

Detailed information is provided in work by Bieniek and Buksa 2024 [49].
